# Gaining the PROMIS perspective from children with nephrotic syndrome: a Midwest pediatric nephrology consortium study

**DOI:** 10.1186/1477-7525-11-30

**Published:** 2013-03-04

**Authors:** Debbie S Gipson, David T Selewski, Susan F Massengill, Larysa Wickman, Kassandra L Messer, Emily Herreshoff, Corinna Bowers, Maria E Ferris, John D Mahan, Larry A Greenbaum, Jackie MacHardy, Gaurav Kapur, Deepa H Chand, Jens Goebel, Gina Marie Barletta, Denis Geary, David B Kershaw, Cynthia G Pan, Rasheed Gbadegesin, Guillermo Hidalgo, Jerome C Lane, Jeffrey D Leiser, Brett W Plattner, Peter X Song, David Thissen, Yang Liu, Heather E Gross, Darren A DeWalt

**Affiliations:** 1 Division of Nephrology, Department of Pediatrics and Communicable Diseases, C.S. Mott Children’s Hospital, University of Michigan, 1500 E Medical Center Drive, SPC5297, Ann Arbor, MI, 48109-5297, USA; 2 Levine Children’s Hospital, Division of Pediatric Nephrology, Charlotte, NC, USA; 3 Department of Biostatistics, School of Public Health, University of Michigan, Ann Arbor, MI, USA; 4 Nationwide Children’s Hospital, The Ohio State University, College of Medicine, Columbus, OH, USA; 5 Pediatric Nephrology, UNC Kidney Center, The University of North Carolina at Chapel Hill, Chapel Hill, NC, USA; 6 Emory University and Children’s Healthcare of Atlanta, Atlanta, GA, USA; 7 University of North Carolina, Chapel Hill, NC, USA; 8 Pediatric Nephrology and Hypertension Division, Children’s Hospital of Michigan, Detroit, MI, USA; 9 Rush Children’s Hospital, Chicago, IL, USA; 10 Division of Nephrology and Hypertension, Cincinnati Children’s Hospital, Cincinnati, OH, USA; 11 Phoenix Children’s Hospital, Phoenix, AZ, USA; 12 Division of Nephrology, The Hospital for Sick Children and University of Toronto, Toronto, ON, USA; 13 Medical College of Wisconsin, Milwaukee, WI, USA; 14 Department of Pediatrics and Center for Human Genetics, Duke University Medical Center, Durham, NC, USA; 15 East Carolina University, Greenville, NC, USA; 16 Feinberg School of Medicine, Northwestern University and Anne & Robert Lurie Children’s Hospital of Chicago, Chicago, IL, USA; 17 Section of Pediatric Nephrology and Hypertension, Riley Hospital for Children at Indiana University Health, Indianapolis, IN, USA; 18 Division of Nephrology, Department of Internal Medicine, University of Michigan, Ann Arbor, MI, USA; 19 Department of Psychology, The University of North Carolina at Chapel Hill, Chapel Hill, NC, USA; 20 Cecil G. Sheps Center for Health Services Research, Division of General Internal Medicine, University of North Carolina, Chapel Hill, NC, USA

**Keywords:** Patient reported outcomes, Quality of life, Nephrotic syndrome, Pediatrics

## Abstract

**Background and objectives:**

Nephrotic syndrome (NS) represents a common disease in pediatric nephrology typified by a relapsing and remitting course and characterized by the presence of edema that can significantly affect the health-related quality of life in children and adolescents. The PROMIS pediatric measures were constructed to be publically available, efficient, precise, and valid across a variety of diseases to assess patient reports of symptoms and quality of life. This study was designed to evaluate the ability of children and adolescents with NS to complete the PROMIS assessment via computer and to initiate validity assessments of the short forms and full item banks in pediatric NS. Successful measurement of patient reported outcomes will contribute to our understanding of the impact of NS on children and adolescents.

**Design:**

This cross-sectional study included 151 children and adolescents 8-17 years old with NS from 16 participating institutions in North America. The children completed the PROMIS pediatric depression, anxiety, social-peer relationships, pain interference, fatigue, mobility and upper extremity functioning measures using a web-based interface. Responses were compared between patients experiencing active NS (n = 53) defined by the presence of edema and patients with inactive NS (n = 96) defined by the absence of edema.

**Results:**

All 151 children and adolescents were successfully able to complete the PROMIS assessment via computer. As hypothesized, the children and adolescents with active NS were significantly different on 4 self-reported measures (anxiety, pain interference, fatigue, and mobility). Depression, peer relationships, and upper extremity functioning were not different between children with active vs. inactive NS. Multivariate analysis showed that the PROMIS instruments remained sensitive to NS disease activity after adjusting for demographic characteristics.

**Conclusions:**

Children and adolescents with NS were able to successfully complete the PROMIS instrument using a web-based interface. The computer based pediatric PROMIS measurement effectively discriminated between children and adolescents with active and inactive NS. The domain scores found in this study are consistent with previous reports investigating the health-related quality of life in children and adolescents with NS. This study establishes known-group validity and feasibility for PROMIS pediatric measures in children and adolescents with NS.

## Introduction

Children with nephrotic syndrome (NS) represent a unique patient population in pediatrics [1]. The signs and symptoms of NS include physical changes typified by edema that can be uncomfortable as well as alarming to patients and families. The disease course that characterizes NS is one of unpredictable relapse and remission, and often treated with multiple courses of corticosteroids, diuretics, antihypertensive medications, and intermittent hospitalizations. In addition to the clinical measures of disease activity, assessment of patient reported outcomes (PRO) and health-related quality of life (HRQOL) can assist in understanding how patients feel and function to better guide clinical care and trials.

A number of disease characteristics inherent to NS including the presence of edema, repeated corticosteroid exposures, and the relapsing nature of NS pose particular challenges to patient HRQOL [2]. Recent studies on the impact of NS on HRQOL have found impairments in social, emotional, and physical functioning [2,3]. The worst cases of NS may progress to end stage renal disease (ESRD). Previous studies in pediatric patients with chronic kidney disease and ESRD have demonstrated a significant burden on HRQOL [4,5]. Studies investigating HRQOL in pediatric chronic kidney disease and NS have utilized a number of different instruments, including but not limited to the ESRD specific PedsQL 3.0™ [6], The Netherlands Organization for Applied Scientific Research Academical Medical Center Child Quality of Life Questionnaire [2], the Child Health and Illness Profile - Adolescent Edition [5], and PedsQL 4.0™ [7]. The diversity of measures used in children younger than 18 years of age makes it difficult to compare research findings across studies and to other patient populations.

The Patient Reported Outcomes Measurement Information System (PROMIS) project was established as part of the National Institutes of Health Roadmap Initiative to create item banks for both adults and children, which are publically available, efficient, precise, and valid across a variety of diseases to assess PROs (http://www.nihpromis.org). In the first phase of PROMIS, 9 item banks were developed for measurement of child self-reported outcomes: depression, anxiety, social-peer relationships, pain interference, fatigue, mobility, upper extremity, anger, and asthma impact [8-13]. PROMIS pediatric measures were developed using qualitative and quantitative methods (focus groups, expert item review, cognitive interviewing, and item administration to a large population of children and adolescents) to create banks of items specific to selected symptoms and quality of life [11,14-17] for use in children 8 to 17 years of age [10,12,13]. PROMIS was developed in order to give researchers flexibility in selecting items or domains that are relevant to the disease of interest. The PROMIS measures have been or are currently under study in several other pediatric chronic health conditions including asthma, sickle cell disease, cancer, rheumatic disease, and obesity. The intent of the PROMIS initiative is to advance the measurement of HRQOL symptoms and functioning by utilizing the same measures across chronic illnesses in children and adolescents, yielding knowledge through comparability of items and scores across diverse pediatric populations. The development and validation of the PROMIS instrument becomes particularly important in pediatric clinical research and pediatric therapeutics as PROs are acceptable clinical trial endpoints to the Food and Drug Administration [18].

This study was designed to evaluate the ability of children and adolescents with nephrotic syndrome to complete the PROMIS assessment via computer and to initiate validity assessments of PROMIS pediatric measures in NS. We hypothesized that the children and adolescents with NS would be able to complete the computer based items and that the PROMIS scores would show worse functioning in children with active NS compared to children without active NS.

## Methods

This cross-sectional study was conducted by the Midwest Pediatric Nephrology Consortium (MWPNC) and included 151 pediatric patients with NS from 16 participating member institutions. Each site obtained individual institutional Institutional Review Board approval. Parents and children gave informed consent and assent respectively, prior to performing the study.

### Training study team members

Personnel at each site (investigators and study coordinators) received web-based training in study procedures; the study operations manual was a reference tool for study conduct, quality control, and recruitment. Ongoing education of site personnel occurred during investigator and coordinator conference calls.

### Eligibility

Consistent with the target age for the PROMIS pediatric item banks, the PROMIS NS cohort study included children 8-17 years. The NS eligibility criteria included presence or history of proteinuria (>2+ urinalysis or urine protein/creatinine ratio >2 g/g) and/or a kidney biopsy that confirmed a NS-inducing condition. Another eligibility criterion was the ability to speak and read English. Exclusion criteria included co-existing medical, psychiatric, or cognitive impairments that would prevent the patient from answering the computer administered questionnaire.

### Study procedures

Both the parent and child completed questionnaires. The parent completed the Family and Medical Information form, which included relationship to child, guardian education level, socioeconomic, and disease specific questions. Child and disease characteristics included: gender, age, race, ethnicity, disease etiology, dialysis, transplant, co-existing conditions, hospitalizations in the previous 6 months (hospitalization history), surgery in the previous 12 months, number of medications, corticosteroid therapy, self-rated edema status, and the guardian’s perception of the child’s weight status (underweight, healthy weight, or overweight). Clinical data such as diagnosis, kidney function represented by estimated glomerular filtration rate (eGFR), chronic kidney disease stage with a higher stage equivalent to poorer kidney function [19], and steroid therapy resistance in NS patients were abstracted from medical records by the local study team. Disease activity was defined as presence of edema at the time of the study visit. Edema was classified as mild or moderate when the feet, ankles, or legs were involved and severe edema was defined to include the abdomen or whole body. Active edema was used in analyses. The GFR was estimated by the classic Schwartz method that was available prior to publication of the updated Schwartz equation [20]. Chronic kidney disease was defined based on estimated GFR (ml/min/1.73 meters squared) as follows; stage I (> 90), stage II (60-89), stage III (30-59), stage IV (15-29), and stage V (< 15 or dialysis dependence).

The children completed the PROMIS pediatric depression, anxiety, social-peer relationships, pain interference, fatigue, mobility, and upper extremity functioning domains using a web-based interface. The definitions of the domains are located at http://www.nihpromis.org/measures/domainframework1. All of these PROMIS items use the context statement “In the past 7 days.” Responses included 5 options ranging from “never” to “almost always” in the majority of domains and from “with no trouble” to “not able to do” for the physical functioning measures. Each PROMIS pediatric domain generates a T-score with a mean of 50 and a standard deviation of 10. The mean of 50 reflects the calibration population and does not represent the general population or other specific group. Higher scores indicate more of the measured symptom, thus signifying worse symptoms of depression, anxiety, fatigue, and pain interference and better functioning for mobility, upper extremity, and peer relationships. These measures have been previously tested in a large group of children and adolescents, confirming their unidimensionality and the extent to which each item is associated with the measured variable (http://www.assessmentcenter.net) [8,10-13]. PROMIS pediatric measures have consistently achieved a reliability of 0.85 or greater over a range of 2 to 4 standard deviations with the short forms [8,10,12,13]. Short forms include 8 items for all domains except fatigue (10 items) and anger (6 items).

In order to decrease the response burden, a sampling plan was devised and is summarized in Table 1.

**Table 1:** PROMIS domain randomization scheme

**Form administered**	Pain interference*	Fatigue*	**Social-peer**	**Depression**	**Anxiety**	**Mobility**	**Upper extremity functioning**
None	50%	50%					
Short form			100%	50%	50%	50%	50%
Full Item bank	50%	50%		50%	50%	50%	50%

*The randomization scheme allocated participants to either the full item bank for the Pain domain or the full item bank for the Fatigue domain.

Participants were randomly assigned to one of two study arms by the PROMIS Assessment Center after they were registered to take the survey. In total, participants completed 70 to 90 questions over 30 to 40 minutes using this strategy.

When full bank data were collected, a short-form score was also calculated. The full item bank and short form domain scores for depression, anxiety, pain interference, fatigue, upper extremity functioning, and mobility were highly correlated, Pearson’s R ranging 0.95-1.0. Consequently, the short form results for all domains are presented here.

### Statistical analysis

Descriptive summary statistics were calculated as frequencies and percentages for demographic and clinical characteristics and as means and standard deviations for eGFR scores and number of medications. Mean scores as well as standard deviations were calculated for each of the 7 domains by full bank and short form. To assess the association of full bank and short-form scores, Pearson’s correlations were calculated where applicable. Known-group validity [21] was assessed for each PROMIS domain by testing whether the scores were different between the two disease activity groups (active vs. inactive). Univariate and multivariate analyses were conducted using the short-form mean scores. Multivariate analysis included covariates, active disease status, age, race, gender, ethnicity, and guardian education level. Mean domain scores were compared by child characteristics and disease status using t-tests for variables with 2 levels and ANOVA for variables with more than 2 levels. Comparisons were performed using non-parametric Wilcoxon test and Kruskal-Wallis test when appropriate for variables that were not normally distributed. Cohen’s d effect sizes were calculated, and interpreted using standard definitions of “small” where absolute effect size (AES) value |d| = 0.0-0.4, “medium” where |d| = 0.5-0.7 and “large” where |d| ≥ 0.8 [22]. Simultaneous regression analyses were conducted for the 7 domain scores.

Although missing clinical data were minimal, two patient reported outcome (PRO) domain scores (Pain Interference and Fatigue) have responses from 50% of patients by design of this study. Note that the other 5 PRO scores are available for the entire cohort. We applied the Expectation-Maximization (EM) algorithm [23] to handle missing outcomes in the simultaneous regression of the 7 PRO scores, which has been implemented in MATLAB package *mvregress*. The EM algorithm is known as a very powerful statistical approach to carrying out statistical analyses with missing data because in this algorithm the distribution of missing data, instead of imputed values, are utilized in the estimation and inference. With the exception of the *mvregress* simultaneous regression analysis, all other statistical analyses were conducted using SAS v9.2.

## Results

Child demographic, disease characteristics, and guardian demographic characteristics are presented in Table 2. There were 151 children with NS enrolled. The majority of guardians who completed the demographic items were parents (n = 138, 91%). Edema was present in 35% (N = 53) of patients, with 32 patients demonstrating mild to moderate edema and 21 patients showing severe edema. Fifty percent (N = 75) of the patients were on steroid therapy at the time of study, and 36% (N=54) of patients required hospitalization in the 6 months prior to the study. Fifty-eight (38%) had steroid sensitive NS, 77 (51%) steroid resistant disease, and steroid resistance status was unknown for 16 (11%). Eighty-eight (58%) patients had CKD stage 1, 36 (24%) had CKD stage II-IV, 7 (5%) had CKD stage V, and 19 (13%) were renal transplant patients. Sixty-five (43%) had other parent-reported health conditions, with asthma (11%), overweight (9%), and premature birth (9%) being the most common.

**Table 2:** PROMIS pediatric nephrotic syndrome cohort

**Child demographics**	***N = 151***
	***n (%)***
Male	79 (52.2)
Age (yrs)	
8-12	62 (41.1)
13-17	89 (58.9)
Race	
White	74 (49.0)
Black	53 (35.1)
Asian	9 (5.9)
Other, Multiple Races	14 (9.3)
Ethnicity	
Non-Hispanic	141 (93.4)
Hispanic	9 (5.9)
Edema Status	
None	96 (63.6)
Mild/moderate	32 (21.2)
Severe	21 (13.9)
eGFR (n), Mean, (SD)**	(n = 144), 95 (47)
Co-Existing conditions (#)	
None	86 (56.9)
One	43 (28.5)
≥ Two	22 (14.6)
Co-Existing conditions:***	
Asthma	17 (11.2)
ADD/ADHD	12 (7.9)
Mental Disorders	11 (7.3)
Overweight	14 (9.3)
Premature birth	14 (9.3)
Rheumatic disease	8 (5.3)
Hospitalization history	
Yes	54 (35.7)
Surgical History	
Yes	43 (28.5)
# of daily medications, Mean(SD))	4.4 (3.4)
Steroid Therapy	
None	74 (49.0)
Alternating day	28 (18.6)
Daily or more than once/day	47(31.1)
**Guardian Demographics**	
Relationship to Child	
Parent	138 (91.4)
Grandparent	5 (3.3)
Guardian or Other	7 (4.6)
Education Level	
< High school	15 (9.9)
High school degree/GED	38 (25.2)
Some college/technical degree	55 (36.4)
College degree or more	42 (27.8)

** eGFR = estimated glomerular filtration rate.

*** Parents reported more than 1 condition; many other conditions in lower frequency (<3%) than listed conditions.

### Feasibility

Four children (2.6%) had missing scores for at least one of the PROMIS domains. Four of 151 (2.6%) ended the survey early, so that measures at the beginning of administration were completed but responses were missing at the end of the administration. Thirty-three of 151 (22%) skipped one or more PROMIS questions within the administration. A missing value analysis examined patterns of missingness by the type of measure administered (full bank or short form), by domain, and by patient age. The domains were administered in random order and no pattern of missingness was observed.

### Descriptive results

The mean scores for the PROMIS domains ranged from 43.9 (fatigue) to 52.5 (upper extremity functioning). There were no differences in the PROMIS measures by gender. However, the PROMIS fatigue domain scores were associated with race (*p* =0.007), and the social-peer relationship scores were worse in children of Hispanic ethnicity compared to those not in the Hispanic ethnic group (*p* = 0.02).

### Validity

As hypothesized, mean scores for several PROMIS domains differed between the NS active and NS inactive groups. Children and adolescents with active NS had significantly worse scores than participants with inactive NS on 4 of the 8 measures, including anxiety, pain interference, fatigue, and mobility (Effect Sizes 0.56-0.79). The two groups did not differ on upper extremity functioning, depression, and social-peer relationships in bivariate analysis (Table 3). An analysis of children who were steroid resistant compared with those who were steroid responsive revealed that the PROMIS Social-Peer Relationships domain was 3 points worse on average in children with steroid resistant NS (*p* = 0.04). The remaining domains were not significantly different.

**Table 3:** PROMIS domain scores by presence of edema for children with NS

	**Overall**		**Edema**				
			**Yes**		**No**		
	**n**	**Mean (SD)**	**n**	**Mean (SD)**	**n**	**Mean (SD)**	**Effect size |d|**
Depression	148	46 (10.8)	52	48 (12.4)	94	45 (9.8)	0.31
Anxiety^*,†^	151	46 (11.5)	53	50 (13.0)	96	44 (9.9)	0.56
Peer Relationships	151	50 (10.4)	53	48 (10.8)	96	51 (10.2)	0.27
Pain Interference^*,†^	82	47 (11.1)	33	51 (10.6)	49	45 (10.9)	0.59
Fatigue^*,†^	68	44 (11.8)	20	50 (13.7)	46	42 (10.1)	0.79
Upper Extremity Functioning	149	53 (7.0)	52	53 (6.9)	95	53 (7.1)	0.01
Mobility^*,†^	150	52 (8.1)	52	49 (9.5)	96	54 (6.9)	0.59

*p < .05 as measured by T-statistic, ^†^*p* < .05 as measured by Wilcoxon test.

### Simultaneous regression models

Regression analysis of all 7 domain scores with covariates of interest, including NS disease activity, gender, age, ethnicity, guardian education, and race, was conducted. This method enables us to borrow information across the PROMIS domain scores. To address the challenge of the 50% administration scheme for 3 domain scores, the EM algorithm was used to derive estimates and p values. Results of the simultaneous regression are reported in Table 4. Overall, 4 PROMIS domains, including anxiety, pain interference, fatigue, and mobility, were sensitive to the marker of active NS. Children with active NS on average had about a 6 point worse score for anxiety and pain interference, a 9 point worse score for fatigue, and a 5 point worse score for mobility (*p* = 0.001, *p* = 0.01, *p* < 0.001, and *p* < 0.001, respectively). There was no evidence for gender differences over the 7 PROMIS domains. Child age was found to be associated with upper extremity functioning scores, such that older children had better upper extremity functioning (*p* < 0.001). Hispanic ethnicity was predictive of worse social-peer relationship (*p* =0.003), upper extremity functioning (*p* = 0.05) and mobility (*p* = 0.03) scores. Children whose guardians had higher education reported lower (better) pain interference scores (*p* = 0.006). Black children reported higher pain interference scores compared with white children (*p* = 0.04). The pain interference scores of Asian children were not different. A sensitivity analysis was performed with the exclusion of children who had CKD stage V or renal transplants. There were 120 patients included in this analysis and the analysis demonstrated no change in the domains that were significant compared to the full model with only minimal changes in the magnitudes of the differences in domain scores (data not shown).

**Table 4:** Results of simultaneous regression model

	**Depression**	**Anxiety**	**Social-peer relationships**	**Pain interference**	**Fatigue**	**Upper extremity functioning**	**Mobility**
	**Estimate (SE)**	**Estimate (SE)**	**Estimate (SE)**	**Estimate (SE)**	**Estimate (SE)**	**Estimate (SE)**	**Estimate (SE)**
Intercept	41 (7.3)	40 (7.3)	61 (6.8)	52 (8.5)	32 (11.6)	45 (4.3)	61 (5.2)
Gender	−1.0 (1.8)	−0.1 (1.8)	1.3 (1.7)	−1.3 (2.2)	−1.5 (2.6)	0.2 (1.1)	−0.1 (1.3)
Current age	−0.2 (0.3)	−0.2 (0.3)	0.2 (0.3)	−0.5 (0.4)	0.3 (0.5)	1.0 (0.2)*	−0.01 (0.2)
Ethnicity	8.3 (5.0)	6.4 (4.9)	−13.3 (4.5)*	6.1 (5.3)	5.6 (8.4)	−5.7 (2.9)*	−7.5 (3.4)*
Active Edema	3.3 (1.9)	6.3 (1.9)*	−3.1 (1.8)	5.6 (2.3)*	9.5 (2.8)*	0.3 (1.1)	−5.0 (1.4)*
Guardian education	−0.5 (1.0)	−1.0 (1.0)	0.3 (0.9)	−3.8 (1.1)*	−0.04 (1.6)	0.1 (0.6)	0.6 (0.7)
Black	−0.3 (2.0)	4.0 (2.0)	−3.1 (1.9)	5.1 (2.4)*	2.6 (3.0)	−0.7 (1.2)	0.1 (1.4)
Asian	0.8 (3.8)	1.2 (3.9)	−3.1 (3.6)	7.5 (4.3)	8.6 (6.2)	−0.9 (2.3)	−2.4 (2.7)
Mixed or Other Race	−0.1 (3.5)	−2.2 (3.6)	1.3 (3.3)	−2.0 (3.9)	9.3 (6.1)	0.3 (2.1)	2.4 (2.5)

*p < .05.

Confirmatory analysis was conducted using data available as collected to evaluate if missing data impacted the results of the simultaneous regression. Simultaneous regression of the 5 PROMIS domain scores completed by all participants had similar results; anxiety and mobility were sensitive to disease activity. In individual regression models for the remaining 2 domains, results were also similar to the simultaneous regression, where disease status was predictive of domain scores for pain interference and fatigue. However, race and ethnicity were not found to be significant predictors of anxiety, upper extremity functioning, mobility, or pain interference.

## Discussion

We report the largest study to date evaluating PRO in children with NS. This North American multi-center study sought to evaluate known-group validity of the PROMIS pediatric measures with a large group of children with NS as an exemplar chronic health condition. Children with NS were able to complete the PROMIS instrument using a web-based interface. This study demonstrated that the PROMIS domains were responsive to NS disease activity using the presence or absence of edema as an indicator of active disease. Children with active NS demonstrated worse functioning in the domains of anxiety, pain interference, fatigue, and mobility when compared to children with inactive disease. These differences were in the domains and expected direction for children affected by active NS.

The PROMIS instruments have been created through a National Institutes of Health initiative to improve the assessment of PROs [24]. Previously, most PRO research instruments utilized classical test theory in their development, but the PROMIS instruments were developed using item response theory [8]. This design allows for broader characterization of a number of domains affected by disease and increased flexibility for researchers to adapt PRO measures to their studies [8]. PROMIS provides researchers with a potentially invaluable tool to measure the impact of pediatric diseases using PROs. A benefit of the PROMIS pediatric measures in clinical trials is the strength of standardized measures in repeated administration that accounts for normative developmental changes while maintaining scores on the same metric. Currently, researchers can use PROMIS to study the impact of disease characteristics and treatments in pediatrics in the PRO domains of depression, anxiety, social-peer relationships, pain interference, fatigue, mobility, upper extremity, and anger as part of any clinical trial [8-13]. In comparison to existing pediatric quality of life measures, PROMIS offers more specific measurement of general health domains, but also the flexibility of using various short forms or computerized adaptive testing that all report on the same metric. For example, as noted in our study, scores using the short forms were nearly identical to scores using the entire item banks. The PROMIS pediatric measures have the additional benefit of being publically available. As part of the pediatric validation process, the PROMIS instruments are currently being studied in asthma, sickle cell disease, cancer, rheumatic disease, obesity, chronic kidney disease, and NS. We present data that show the PROMIS instruments detect the impact of NS activity on pediatric patients.

The long-term impacts of childhood kidney disease have been addressed in longitudinal studies involving children. In children initiating dialysis, psychological problems have been identified and often persist into adulthood [25,26]. Recent studies have provided some insight into the fact that children with NS are vulnerable to challenges in their HRQOL in areas including physical, school, emotional, and social functioning [2,3]. Ruth and colleagues showed that severity of illness in steroid responsive NS as determined by steroid dependence or cyclophosphamide treatment was associated with worse physical and emotional well-being [2]. These studies were relatively small in nature. We extend these by reporting the largest multi-center study to date evaluating PROs in children with NS. Previous studies focused on chronic disease characteristics, but have not evaluated the impact of acute disease activity (edema) that may provide specific burden to patients’ HRQOL in NS. We provide data showing that the PROMIS instrument was sensitive to disease activity, specifically edema, on the PROs that previous research has demonstrated are affected by NS. We show that children with active NS have significantly worse scores in the domains of pain interference, anxiety, fatigue, and mobility relative to those children in remission.

Edema represents one of the most visibly evident symptoms of NS. Patients with NS are prone to edema as their disease process relapses or proves resistant to available therapies. In those with NS, edema is often the only overt sign of disease activity, but does not necessarily reflect disease progression. The impact of edema on patients includes altered physical appearance, mobility challenges, and pain. The PROMIS instrument yielded PRO data that are consistent with previous reports showing that children with NS are vulnerable in the areas of emotional well-being and mobility [2,3,5,7]. Previous reports have not evaluated the impact of specific markers of disease activity such as edema and their impact on PRO data. We extend the literature by demonstrating that patients with active NS have an increased burden in the domains of pain interference, anxiety, fatigue, and mobility relative to children in remission. This finding is strengthened by the fact that our comparative patient population was children with inactive NS, whereas previous studies utilized healthy controls. It is understandable that children with active NS would have issues with pain interference, fatigue, and mobility as the presence of edema can directly contribute to these physical attributes. The increased burden of anxiety symptoms may reflect the loss of disease control in the presence of edema. Conversely, the domains of depression, social-peer relationships, and upper extremity functioning were not different between the disease activity strata.

The PROMIS measures performed as we had anticipated in their ability to distinguish differences in PROs in individuals with active NS. Furthermore, our observed relationships between patient demographics and PROs are consistent with previous published reports in children with kidney disease and other chronic diseases. We describe that gender does not influence the outcomes of PROs in children with NS. This is consistent with previous reports and the recent findings of Gerson et al in their descriptions of children with mild to moderate chronic kidney disease [7,27], indicating the strength of the instrument. Our findings regarding the pain interference domain are consistent with a recent investigation into health-related quality of life utilizing the PROMIS instrument in children with cancer that showed Black children had an increased burden of pain interference [28]. This finding has not been previously described in children and adolescents with NS.

A potential limitation of this study is that it utilized an internal comparative patient population rather than healthy controls. However, this design allowed us to demonstrate the strength of the PROMIS instrument in that it can detect clinically meaningful differences within a patient population with a range of disease severity. The PROMIS pediatric self-report instruments are limited to ages 8-17. A parent proxy-report instrument is currently being validated for ages 5 to 17. At the time of this study, only an English version of the PROMIS pediatric instruments was available. Consequently, this study included only children with English literacy. Lastly, this study was cross-sectional and does not document longitudinal changes. A longitudinal study validating the responsiveness of the PROMIS measures in patients with NS during episodes of relapse and remission is currently underway.

Specific strengths of this study include the inclusion of patients from a wide variety of participating centers across North America. This represents one of the broadest patient samplings studying PROs in children with NS and supports the potential generalizability of these results.

This study suggests possible strengths and limitations of the PROMIS instrument for children with NS. The instrument allows the assessment of factors that are important from the patient perspective but which may not receive due attention in the research or office setting. The availability of the instrument in a web-based format allows the collection of data in home and office settings for ease of administration. Auto scoring functions may facilitate the timely review and integration of the most concerning areas if implemented within the clinical care environment. This study suggests that domains of anxiety, pain, fatigue, and mobility but not depression change with disease status. This study also demonstrates the consistent results from long or short forms. As the PROMIS measures were developed to allow the selection of domains of relevance to a particular population, the number of domains and items selected for use in research or clinical settings can be tailored to minimize response burden. Limitations of the instrument most relevant to pediatric NS are the minimum age for self-report of 8 years and a lack of multiple language versions (at this time, PROMIS has English and Spanish versions only). Incident pediatric NS affects all ages but a disproportionate number affects children under the age of 5 years. The absence of self-report below age 8 years and absence of parent proxy report for children less than 5 years is a gap that requires attention if PROMIS is to gain widespread implementation across the affected population.

## Conclusion

Children and adolescents with NS were able to successfully complete the PROMIS measures using a computer-based interface in an outpatient setting. The PROMIS pediatric instruments were sensitive to differences in clinical status in the largest multi-center study to date of children with NS in North America to date. Specifically, we demonstrated that children with active NS had an increased burden in the domains of pain interference, anxiety, fatigue, and mobility relative to children in remission. The next step in the PROMIS validation process for children will be to evaluate self- and parent-proxy report PROMIS in a longitudinal study and to quantify the minimally important differences in change in PROMIS scores in patients with NS.

## Abbreviations

PRO: Patient reported outcomes; PROMIS: Patient reported outcomes measurement information system; NS: Nephrotic syndrome; ESRD: End stage renal disease; HRQOL: Health-related quality of life; eGFR: estimated glomerular filtration rate

## Competing interest

The authors declare that they have no competing interests. The authors have no financial relationships or conflicts of interest relevant to this article to disclose.

## Authors’ contributions

DTS, LW, BWP, DSG, KLM, DAD, HEM, DT: Drafting the manuscript revising it critically for important intellectual content. DSG, SFM, EH, CB, MEF, JDM, LAG, JM, GK, DHC, JG, GMB, DG, DBK, CGP, RG, GH, JL, JDL,: Substantial contributions to the acquisition of data and execution of the study at the multiple sites including site principle investigators. DSG, KLM, JM, DT, YL, HEG, DAD: Substantial contributions to conception and design. Design and execution of the study, scoring of the PROMIS instruments, analysis of the PROMIS scores, and interpretation of data. Drafting the article or revising it critically for important intellectual content. All authors read and approved the final manuscript.
